# AKiPal: Protocol for a Mixed-Methods Pilot and Feasibility Study of a Nurse-Led Acupressure Intervention in Palliative and Hospice Care on Symptom Burden and Body Awareness

**DOI:** 10.3390/healthcare14162493

**Published:** 2026-08-11

**Authors:** Melanie Joshi, Mareike Loebberding, Eva Bothe, Lisanne Herzhoff, Julia Schmidtke, Raymond Voltz, Julia Strupp

**Affiliations:** 1 Department of Palliative Medicine, Faculty of Medicine and University Hospital, University of Cologne, 50937 Cologne, Germany; melanie.joshi@uk-koeln.de (M.J.); mareike.loebberding@uk-koeln.de (M.L.); eva.bothe@uk-koeln.de (E.B.); lisanne.herzhoff@uk-koeln.de (L.H.); julia.schmidtke@uk-koeln.de (J.S.); raymond.voltz@uk-koeln.de (R.V.); 2 Center for Integrated Oncology Aachen Bonn Cologne Duesseldorf (CIO ABCD), Faculty of Medicine and University Hospital, University of Cologne, 50937 Cologne, Germany; 3 Center for Health Services Research, Faculty of Medicine and University Hospital, University of Cologne, 50933 Cologne, Germany

**Keywords:** acupressure, body awareness, complementary medicine, feasibility study, hospice care, palliative care, qualitative research, quantitative research, symptom burden

## Abstract

**Background:** Patients receiving palliative and hospice care frequently experience high symptom burden alongside altered body perception due to progressive disease and physical decline. While acupressure has demonstrated beneficial effects on symptoms such as nausea, pain, fatigue, and anxiety, its potential influence on body awareness has not been systematically investigated. Furthermore, evidence regarding the feasibility and implementation of nurse-led acupressure interventions across different palliative and hospice care settings remains limited. This study aims to explore the feasibility, acceptability, and potential effects of acupressure on symptom burden and body awareness in palliative care settings. **Methods:** This pilot study evaluates a nurse-led acupressure intervention aimed at improving body awareness in patients receiving palliative and hospice care across specialized inpatient and outpatient settings. This study is designed as a prospective, exploratory, mixed-methods pilot and feasibility study using a pre-post design across multiple care settings, including a palliative care unit, specialized palliative home care (SAPV), an inpatient palliative consultation service (PMD), and a hospice. A total of 60–80 adult patients with incurable illnesses will be recruited. The intervention consists of standardised acupressure administered by trained nursing staff, with at least two sessions per participant. Primary outcomes focus on feasibility and acceptability indicators, including recruitment, retention, adherence, intervention documentation, and data completeness. Exploratory clinical outcomes include symptom burden, body awareness, and use of on-demand medication. Quantitative outcomes include symptom burden (MIDOS), body awareness (State Mindfulness Scale—Body subscale), and use of on-demand medication. Qualitative data will be collected through brief patient interviews using the Body Image Assessment (BIA) and semi-structured interviews with patients and healthcare providers after the completed intervention. Data will be analysed using descriptive and exploratory inferential statistics, alongside a linguistic and thematic analysis. Quantitative and qualitative findings will be integrated using a convergent triangulation approach. **Discussion:** This study will provide first insights into the feasibility and acceptability of acupressure in palliative and hospice care and generate hypotheses regarding its potential effects on symptom relief and body awareness. Findings will inform the design of a future randomized controlled trial and contribute to the implementation of complementary therapies in integrative palliative care.

## 1. Introduction

Palliative and hospice care aim to improve the quality of life for patients with life-limiting illnesses by addressing physical, psychological, social, and existential needs. Despite advances in symptom management, many patients continue to experience substantial symptom burden, including pain, fatigue, nausea, dyspnoea, and anxiety, particularly in the last year of life [1,2]. In addition to these symptoms, patients frequently undergo profound physical changes such as cachexia, oedema, and functional decline, which can significantly alter body perception and body image [3].

Body awareness has increasingly become an important construct in health-related research because it reflects how individuals perceive and attend to internal bodily sensations. Although body awareness, body image, and body perception are closely related concepts, they describe different aspects of bodily experience. Body awareness primarily refers to attention to and awareness of internal bodily sensations and interoceptive processes [4]. Body image encompasses cognitive, emotional, and perceptual attitudes toward one’s own body and includes predominantly exteroceptive and visual aspects of bodily experience. Body perception is considered a broader multidimensional construct that integrates physical, psychological, emotional, and social dimensions of experiencing the body [4].

In palliative care, progressive disease, physical decline, treatment-related changes, and increasing dependency frequently alter all of these dimensions. Patients often experience changes in their relationship with their body, which may influence symptom perception, emotional well-being, identity, and quality of life. Because the present study investigates the effects of a touch-based intervention, it focuses specifically on body awareness, defined as awareness of internal bodily sensations and states, as this construct may be particularly relevant for understanding mind–body interactions during advanced illness.

Disturbances in body image have been associated with increased symptom burden, including pain, fatigue, anxiety, and depression, as well as reduced well-being and impaired social interaction [5,6]. Qualitative research additionally highlights patients’ experiences of loss of identity, diminished self-worth, and challenges in interpersonal relationships related to bodily changes [7]. These findings suggest that bodily experience extends beyond physical symptoms and represents an important component of holistic palliative care. Consequently, interventions that support patients in reconnecting with their bodily experience may contribute not only to symptom management but also to emotional adaptation and overall well-being.

Complementary approaches are increasingly used in palliative care to support symptom management and enhance well-being. Among these, acupressure—a non-invasive technique derived from traditional Chinese medicine involving manual pressure on specific body points—has shown promising results. Current evidence indicates that acupressure may reduce nausea, pain, and anxiety, and improve quality of life in patients with advanced disease [8,9]. For example, studies have demonstrated significant reductions in pain intensity and analgesic use, as well as improvements in fatigue and gastrointestinal symptoms [10,11,12]. However, although these results are promising, the current evidence remains limited and heterogeneous, and further high-quality studies are warranted. Clinical guidelines, such as the German S3 guideline for complementary medicine in oncology, recommend acupressure as a supportive intervention [13].

In addition to symptom relief, touch-based and mind–body interventions may influence patients’ awareness of bodily sensations and their relationship with their body. Body awareness is closely linked to symptom perception, emotional regulation, and overall well-being [7]. Because acupressure involves focused tactile stimulation and attention to bodily sensations, it may particularly influence body awareness and patients’ subjective experience of their body. Interventions that enhance awareness of bodily sensations may support patients in coping with illness-related changes and improve their subjective experience of their body. Qualitative studies in hospice settings suggest that complementary therapies can promote a sense of holistic well-being, including physical, emotional, and spiritual dimensions, and may enhance body awareness through therapeutic touch and patient engagement [14].

However, despite these promising findings, research on acupressure in palliative care has primarily focused on symptom control. The potential relationship between acupressure and body awareness has not been systematically investigated. This represents a significant gap, particularly given the strong association between body perception and symptom burden.

Moreover, the current evidence base in Germany regarding the implementation of complementary therapies in palliative and hospice care remains limited and heterogeneous. Most studies focus on specific patient groups, particularly those with cancer, while less attention has been given to patients with non-malignant diseases. In addition, there is a lack of research examining implementation processes, feasibility, and acceptability across different care settings. Given that acupressure can be considered a complex intervention—comprising multiple interacting components such as standardised application, patient engagement, and contextual adaptation—there is a need for exploratory, multimethod studies to assess its feasibility and potential effects in real-world settings.

Acupressure can be regarded as a complex intervention because its effects are likely to result from multiple interacting components rather than from manual pressure alone. These components include standardised application techniques, therapeutic touch, patient engagement, communication between patients and healthcare professionals, and adaptation to different clinical environments. Consequently, understanding both clinical outcomes and implementation processes is essential before evaluating effectiveness in a definitive randomized controlled trial.

Nurse-led palliative care interventions may improve patient-centred outcomes when implemented as comprehensive and contextually integrated care models. However, further research is needed to standardise intervention approaches, evaluate implementation in routine practice, and broaden the evidence base beyond oncology populations [15]. Evidence on nurse-led delivery of complementary interventions in palliative and hospice care, such as acupressure, remains limited.

Because acupressure represents a complex intervention involving therapeutic touch, patient engagement, and delivery across diverse palliative care settings, a mixed-methods approach was chosen. Quantitative measures will explore changes in symptom burden and body awareness, whereas qualitative data will provide insight into patients’ experiences, acceptability, and implementation. Together, these findings will inform the design of a future randomized controlled trial. The present study addresses this gap by investigating the feasibility, acceptability, and potential effects of a nurse-led acupressure intervention on symptom burden and body awareness in palliative and hospice care across multiple settings. By integrating quantitative and qualitative findings, the study aims to generate hypotheses regarding potential mechanisms of action, evaluate implementation processes, and provide the methodological foundation for a future randomized controlled trial.

## 2. Materials and Methods

This study protocol is reported in accordance with the Standard Protocol Items: Recommendations for Interventional Trials (SPIRIT) guidelines, which provide recommendations for the minimum content of trial protocols to ensure transparency and completeness. The study itself is additionally informed by the RE-AIM framework (Reach, Effectiveness, Adoption, Implementation, and Maintenance) to support the evaluation of feasibility, implementation processes, and potential transferability into routine palliative and hospice care practice [16,17].

The primary aim of this study is to assess the feasibility and acceptability of a standardised acupressure intervention in palliative and hospice care, and to explore its potential effects on symptom burden and body awareness. This study is designed as a prospective, exploratory, multimethod pilot and feasibility study using a pre–post design. A mixed-methods approach was selected because feasibility studies of complex interventions require evaluation of both measurable outcomes and implementation processes. Quantitative data will provide preliminary estimates of changes in symptom burden and body awareness, whereas qualitative findings will contribute to understanding participants’ experiences, intervention acceptability, and contextual factors influencing implementation across different palliative care settings. The intervention is conceptualized as a complex intervention, according to the Medical Research Council framework, as it involves multiple interacting components, including standardised delivery of acupressure, active patient engagement, and contextual adaptation across different palliative and hospice care settings [18]. Complex interventions are characterized by interactions between their components, the influence of context on delivery and outcomes, and multiple potential mechanisms of effect, rather than a single linear causal pathway.

Given this complexity, a feasibility and acceptability study is an appropriate first step to assess intervention delivery, acceptability, and preliminary signals of effectiveness, and to inform the design and refinement of a future definitive randomized controlled trial. The RE-AIM framework will further guide the assessment of intervention reach, implementation, adoption within clinical settings, and perceived sustainability in routine care.

The acupressure sessions will be delivered by trained nurses, making this a nurse-led intervention that is fully integrated into routine palliative and hospice care practices.

### 2.1. Study Setting

The study will be conducted in multiple care settings affiliated with the Department for Palliative Medicine at the University Hospital Cologne, including an inpatient palliative care unit, a palliative consultation service within acute hospital wards, specialized outpatient palliative care (SAPV), and an inpatient hospice. This multi-setting approach allows for the evaluation of feasibility and implementation across diverse real-world contexts.

The total study duration is 24 months, comprising a preparatory phase (months 1–3), an 18-month recruitment and intervention phase (months 4–18), and a data analysis and dissemination phase (months 19–24).

### 2.2. Participants and Recruitment

Eligible participants are adult patients (≥18 years) with incurable, life-limiting illnesses receiving care in one of the participating settings. Inclusion criteria require the presence of at least one clinically relevant symptom (e.g., pain, fatigue, nausea, dyspnoea, anxiety), reflecting typical symptom burden in palliative care populations, as well as the ability to provide informed consent. The predominant symptom(s) prompting participation will be documented at baseline to characterize the study population and to support exploratory analyses of symptom-specific responses to the intervention. Symptoms prompting each acupressure session will be documented descriptively. Patients should have an expected duration of care sufficient to allow at least two acupressure sessions, typically five to seven days.

Exclusion criteria include severe cognitive impairment or altered mental status precluding participation, as well as medical contraindications to acupressure, such as skin lesions, infections, fractures, or other conditions affecting the relevant body areas.

Recruitment will be conducted by treating physicians and nursing staff in collaboration with the study team. Potential participants will receive detailed verbal and written information about the study, and written informed consent will be obtained prior to enrolment. Participation is voluntary, and withdrawal is possible at any time without affecting ongoing care.

### 2.3. Sample Size Justification

As a pilot and feasibility study, this trial is not powered to detect statistically significant differences in clinical outcomes. Instead, the sample size is based on recommendations for feasibility studies and pragmatic considerations related to recruitment capacity across settings. Recruitment will be monitored across all participating settings to ensure representation from inpatient palliative care, specialized outpatient palliative care, the palliative consultation service, and hospice care. Recruitment numbers from each setting will be reported descriptively as feasibility outcomes.

A total of 60 to 80 participants is planned. This sample size is based on recommendations for pilot and feasibility studies rather than statistical power calculations. It is considered sufficient to estimate recruitment and retention rates, intervention adherence, data completeness, variability of outcome measures, and preliminary effect sizes to inform sample size calculations for a future randomized controlled trial.

### 2.4. Intervention

The intervention consists of standardised acupressure and is nurse-led, delivered by trained nursing staff within routine palliative and hospice care settings. Prior to study initiation, all participating nurses will undergo a structured two-day training program led by an experienced instructor with qualifications in palliative care and acupressure. The training includes theoretical foundations, indications and contraindications, and supervised practical exercises tailored to the specific patient population. To support consistent application, practical competency will be reviewed at the end of the training. Intervention fidelity will be supported through the use of a standardised intervention manual, structured documentation forms, and regular communication between the study team and participating nurses. Intervention documentation will be reviewed periodically to identify protocol deviations and to ensure consistent delivery across study sites.

Acupressure will be delivered according to a predefined protocol using standardised acupressure points selected for their relevance to common symptoms in palliative care. Each session will last approximately 20 mins, with allowable deviations of ±5 min documented. Each participant will receive at least two sessions during the study period.

Where appropriate and desired, relatives or other family caregivers may additionally receive brief instruction in simple standardised acupressure techniques to support involvement in care. They will receive an illustrated instruction manual describing the standardised procedure and relevant acupressure points. The optional involvement of family caregivers is based on the non-invasive nature of acupressure and the use of standardised, clearly described techniques that can be applied following written instruction. Participants may also receive an acupressure seed intended for temporary self-administered stimulation for up to 12 h.

A detailed intervention manual will guide all aspects of delivery, including acupressure-point selection, duration, frequency, and documentation procedures. Each session will be documented with respect to timing, duration, use of acupressure seeds, patient responses, and any deviations from the protocol. Any adverse events, such as local discomfort or circulatory reactions, will be systematically recorded and medically evaluated if necessary. In inpatient settings, intervention frequency may be influenced by the patient’s clinical condition and routine care processes. Reasons for missed or postponed sessions, including patient deterioration or organizational constraints, will be documented.

### 2.5. Outcome Measures and Data Collection

**Figure 1:**
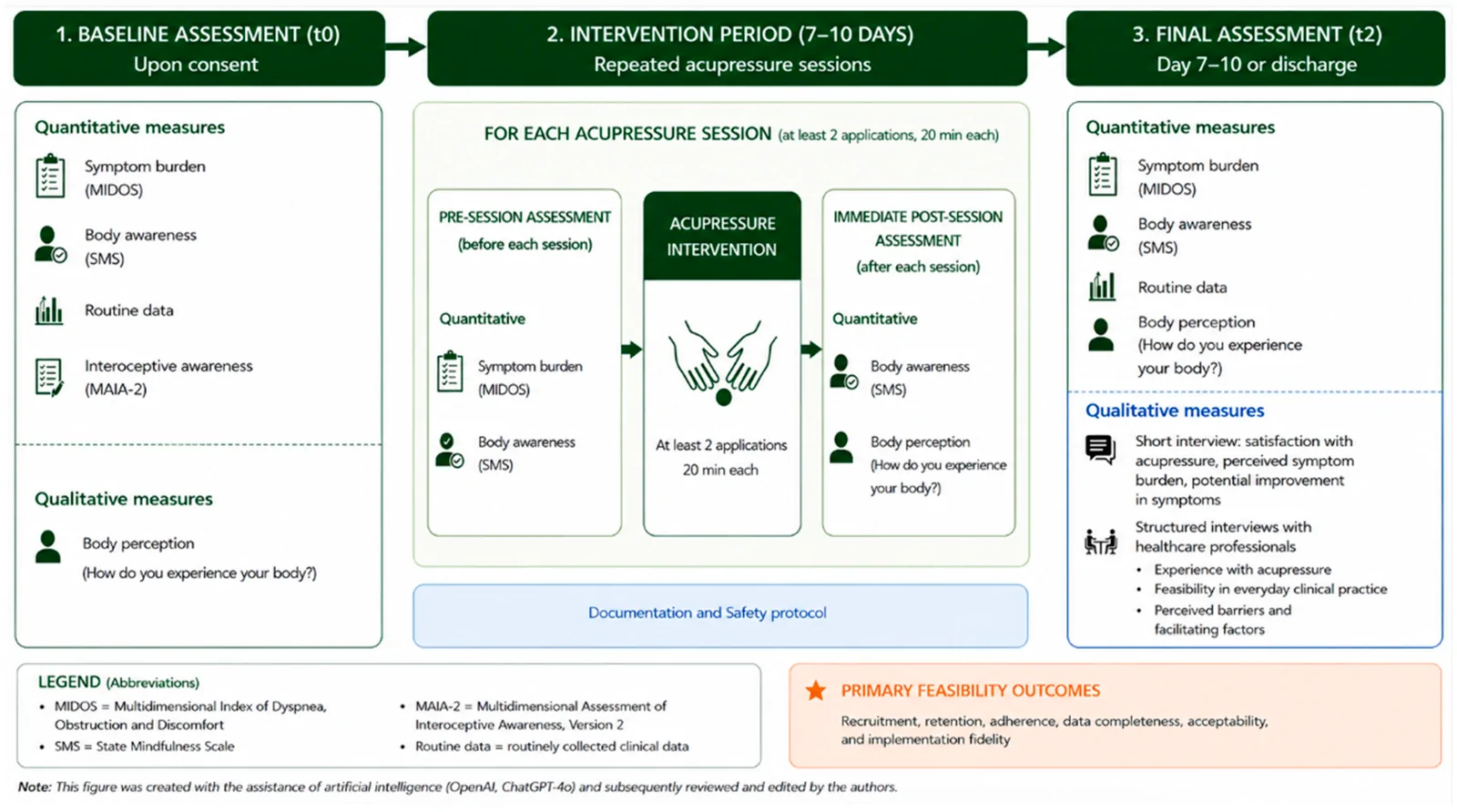
Intervention overview.

Data are collected prospectively throughout each participant’s study period. In addition to the baseline assessment, symptoms, body awareness, and body perception are assessed at predefined application-related intervals immediately before and after each acupressure session to capture short-term responses to the intervention. The primary evaluation follows a pre-post design, with assessments conducted at baseline (day 0) and after the intervention period (typically day 7–10 or at discharge, whichever occurs first). Both quantitative and qualitative data are collected to allow for a comprehensive evaluation (see Figure 1).

Quantitative outcomes include symptom burden, measured using the Minimal Documentation System (MIDOS) [19], a validated instrument for assessing symptom burden in palliative care, and body awareness, assessed using the Body subscale of the State Mindfulness Scale (SMS) [20], which assesses momentary body awareness, together with selected items from the Multidimensional Assessment of Interoceptive Awareness (MAIA-2), which assesses interoceptive body awareness [21]. In addition, the use of on-demand medication is extracted from routine clinical records. Sociodemographic and clinical variables are recorded at baseline to characterize the study population and explore potential confounding factors.

Qualitative data are collected through brief, semi-structured interviews with patients, focusing on subjective body awareness and experiences with acupressure. To minimize burden in this vulnerable population, interviews are designed to be concise and focused. In addition, semi-structured interviews with healthcare professionals are conducted to explore feasibility, acceptability, and contextual factors influencing implementation.

### 2.6. Implementation Outcomes

The primary outcomes of this pilot study are feasibility and acceptability. Feasibility will be assessed through indicators such as recruitment and retention rates, adherence to the intervention protocol, completeness of data collection, time requirements, and integration into routine care processes. Acceptability will be evaluated from both patient and provider perspectives, including perceived usefulness, satisfaction, and willingness to continue or recommend the intervention.

These outcomes will be assessed using a combination of standardised questionnaires, qualitative interviews, and analysis of intervention documentation.

### 2.7. Data Analysis

Quantitative data will be analysed using descriptive statistics to summarize sample characteristics, feasibility indicators, and outcome measures. Exploratory estimation analyses will be conducted to examine pre–post changes in symptom burden and body awareness, using paired t-tests or non-parametric equivalents depending on data distribution, with effect sizes and corresponding 95% confidence intervals reported where appropriate. Effect sizes will be calculated to inform future sample size planning. Subgroup analyses across care settings and according to the use of acupressure seeds will be conducted where feasible, and potential confounding variables will be considered in sensitivity analyses.

Qualitative data (Body Image Assessment; semi-structured interviews) will be audio-recorded, transcribed verbatim, and analysed using the Różańska´s linguistic approach and thematic analysis following Braun and Clarke. Coding will be performed independently by two researchers, with discrepancies resolved through discussion to ensure analytical rigor and credibility.

Integration of quantitative and qualitative findings will be achieved using a convergent triangulation approach. Quantitative and qualitative data will first be analysed separately and subsequently integrated by comparing areas of agreement, complementarity, and divergence. This mixed-methods approach enables a more comprehensive understanding of both outcome-related effects and contextual factors influencing implementation, thereby supporting hypothesis generation and informing the design of future randomized controlled trials.

### 2.8. Participant Timeline

Following eligibility screening and informed consent, participants will undergo baseline assessment (day 0), including collection of sociodemographic and clinical data, as well as baseline measures of symptom burden and body awareness.

The intervention phase takes place over approximately five to seven days, during which participants receive at least two acupressure sessions. This timeframe was considered feasible based on clinical routines and the expected availability of patients for repeated assessments and was adopted as a pragmatic approach across the participating settings, including inpatient palliative care, SAPV, hospice care, and palliative consultation services. However, the individual study period may be shorter or longer depending on the patient’s clinical condition, care setting, length of stay, and timing of intervention delivery.

Documentation will include confirmation of intervention delivery by the treating nurse, duration of the session, patient tolerance, and reasons for any deviations from the planned intervention schedule. Post-intervention assessment is planned between day 7 and day 10 or at discharge, whichever occurs first. This includes repeated quantitative measures and qualitative assessment of patient experiences. A subset of healthcare professionals will be interviewed after the intervention phase to assess implementation outcomes.

### 2.9. Ethics, Safety and Data Protection

Ethical approval has been obtained from the Ethics Committee of the Medical Faculty of the University of Cologne prior to study initiation (#26-1150). All participants will provide written informed consent.

Patient safety will be monitored throughout the study. Any adverse events related to the intervention will be documented and managed appropriately. If a participant loses the capacity to consent during the study, participation will be discontinued, and previously collected data will be handled in accordance with ethical and legal requirements.

All data will be handled confidentially and in compliance with the General Data Protection Regulation (GDPR). Personal data will be pseudonymized, and access will be restricted to authorized members of the research team.

## 3. Discussion

This study protocol describes a mixed-methods pilot and feasibility study investigating the implementation of a nurse-led acupressure intervention and its potential effects on symptom burden and body awareness in palliative and hospice care. By combining quantitative and qualitative approaches across multiple care settings, the study aims to generate a comprehensive understanding of both outcome-related changes and contextual factors influencing the delivery of this complex intervention. This mixed-methods design is particularly appropriate for evaluating complex interventions because it enables the simultaneous assessment of feasibility, preliminary clinical outcomes, and implementation processes.

A key contribution of this study is its focus on embodied experience in palliative care, particularly body awareness. While previous research on acupressure and other complementary approaches has primarily concentrated on symptom relief, less attention has been given to how patients experience and relate to their bodies in the context of advanced illness. This is noteworthy, as progressive disease often alters bodily perception, emotional processing, and identity. Approaches derived from complementary and mind–body practices, including acupressure, increasingly emphasize not only symptom modulation but also attentional engagement with bodily sensations, which conceptually overlaps with mindfulness-based processes and interoceptive awareness. Exploring this dimension may therefore extend current understandings of holistic, patient-centred care at the end of life.

The inclusion of both standardised measures and qualitative data allows for a nuanced assessment of this construct, which is particularly relevant in a population with heterogeneous needs and experiences.

The nurse-led nature of the intervention represents an additional strength. Embedding acupressure within routine nursing care reflects real-world applicability and supports the integration of low-threshold complementary interventions into standard palliative care practice. Importantly, nurse-led delivery may also facilitate patient–provider interaction and guided attention to bodily sensations, which could contribute to enhanced bodily self-awareness in patients. This may be particularly relevant in palliative care, where nursing staff play a central role in providing continuity, reassurance, and therapeutic presence. In this context, acupressure can be understood not only as a symptom-oriented technique but also as a structured form of therapeutic touch that may support relaxation, interoceptive attention, and embodied awareness.

Another strength is the mixed-methods design, which enables the integration of quantitative outcomes with qualitative insights into patient and provider perspectives. This approach is particularly suited to evaluating complex interventions in real-world settings, where feasibility, acceptability, and contextual influences are critical determinants of successful implementation. The convergent triangulation of quantitative and qualitative findings will allow complementary interpretation of measurable outcomes and participants’ experiences, thereby providing a richer understanding of how and under which circumstances the intervention may be beneficial. The inclusion of multiple care settings—including inpatient, outpatient, and hospice care—enhances the relevance and transferability of the findings to real-world clinical practice and allows for the identification of setting-specific facilitators and barriers to implementation.

At the same time, several limitations should be considered. As a pilot and feasibility study, the sample size is limited and not powered to detect statistically significant effects. The pre–post design without a control group restricts causal inference, and observed changes may be influenced by confounding factors such as disease progression, concurrent treatments, or contextual variables. Additionally, the heterogeneity of the study population, while reflecting real-world practice, may introduce variability that complicates interpretation of outcomes. The use of a body awareness measure that has not yet been specifically validated in palliative care populations represents another limitation; however, this study provides an opportunity to explore its applicability and inform future instrument development. Furthermore, the integration of qualitative interview data through convergent triangulation may help contextualize and interpret quantitative findings related to body awareness, thereby partially mitigating this methodological limitation.

These limitations underline that the present study is designed to evaluate feasibility, acceptability, and preliminary signals of potential effects rather than to establish intervention efficacy. The findings should therefore be interpreted as hypothesis-generating and will inform the design of future controlled studies.

Variability in intervention delivery across different clinical settings and fluctuations in patient condition are inherent challenges in palliative care research and will be considered when interpreting feasibility outcomes. These include variability in patient condition and length of stay, potential time constraints for nursing staff, and the integration of the intervention into routine care processes. To address these challenges, the study incorporates structured training, a standardised intervention protocol, and ongoing documentation of implementation processes. The evaluation of feasibility and acceptability will provide valuable information on how such interventions can be sustainably integrated into clinical practice.

Despite these limitations, the study has important implications. It will provide preliminary data on recruitment, retention, adherence, intervention fidelity, and outcome variability, which are essential for planning a future randomized controlled trial. Furthermore, it will generate hypotheses regarding the relationship between acupressure, symptom burden, and body awareness, and identify potential mechanisms of action. The qualitative findings will contribute to a deeper understanding of patient experiences and inform the refinement of both the intervention and study design.

In addition to its scientific contribution, the study has practical relevance for integrative palliative care. By systematically evaluating a low-threshold, non-pharmacological intervention that can be delivered by nursing staff, the study addresses the need for accessible approaches to symptom management and holistic care. The planned development of a practice-oriented factsheet will support knowledge translation and provide guidance for clinicians seeking to implement complementary therapies in palliative and hospice settings.

In conclusion, this pilot and feasibility study represents an important step towards the systematic evaluation of acupressure as part of integrative palliative care. Beyond providing preliminary evidence regarding feasibility and acceptability, it will contribute to understanding how nurse-led complementary interventions can be implemented across diverse palliative care settings and inform the design of a future adequately powered randomized controlled trial.

## 4. Conclusions

This pilot and feasibility study provides an initial evidence base for the implementation of nurse-led acupressure in palliative and hospice care. It will generate critical insights into feasibility, acceptability, and outcome variability, as well as exploratory signals regarding the relationship between acupressure, symptom burden, and body awareness. These findings will inform the design of a future randomized controlled trial and contribute to the development of scalable, nurse-delivered integrative interventions in palliative care.

## Data Availability

The datasets generated and/or analysed during the current study are not publicly available due to the sensitive nature of palliative care data and the potential risk of compromising participant privacy. Data may be available from the corresponding author on reasonable request.

## Abbreviations

The following abbreviations are used in this manuscript:

BIA	Body Image Assessment
GDPR	General Data Protection Regulation
MIDOS	Minimal Documentation System
PMD	Palliative inpatient care consultation service
RE-AIM	Reach, Effectiveness, Adoption, Implementation, Maintenance
RCT	Randomized controlled trial
SAPV	Specialized palliative home care
SMS	State Mindfulness Scale
SPIRIT	Standard Protocol Items: Recommendations for Interventional Trials
AKiPal	Acupressure in palliative and hospice care
